# Keystone microalgae species determine the removal efficiency of sulfamethoxazole: a case study of *Chlorella pyrenoidosa* and microalgae consortia

**DOI:** 10.3389/fpls.2023.1193668

**Published:** 2023-07-05

**Authors:** Ruohan Huang, Wan Liu, Jinghua Su, Shihao Li, Liqing Wang, Erik Jeppesen, Wei Zhang

**Affiliations:** ^1^ Key laboratory of Exploration and Utilization of Aquatic Genetic Resources of the Ministry of Education, Engineering Research Center of Environmental DNA and Ecological Water Health Assessment, Shanghai Ocean University, Shanghai, China; ^2^ Research Institute of Natural Ecology Conservation, Shanghai Academy of Environmental Sciences, Shanghai, China; ^3^ Shanghai Aquatic Technology Co., Ltd, Shanghai, China; ^4^ Department of Ecoscience, Aarhus University, Aarhus, Denmark; ^5^ Sino-Danish Centre for Education and Research (SDC), University of Chinese Academy of Sciences, Beijing, China; ^6^ Limnology Laboratory and EKOSAM, Department of Biological Sciences, Middle East Technical University, Ankara, Türkiye; ^7^ Institute of Marine Sciences, Middle East Technical University, Mersin, Türkiye

**Keywords:** sulfamethoxazole, microalgae consortia, diversity, ecotoxicity, removal

## Abstract

In recent years, antibiotics pollution has caused serious harm to the aquatic environment, and microalgae mediated degradation of antibiotics has attracted increasing attention. However, the potential toxicity of antibiotics to keystone microalgae species or their microalgae consortia, and the impact of microalgal diversity on antibiotic removal need to be further studied. In this study, we investigated the removal efficiency and tolerance of five freshwater microalgae (*Chlorella pyrenoidosa*, *Scenedesmus quadricauda*, *Dictyosphaerium* sp., *Haematoccocus pluvialis*, and *Botryococcus braunii*) and their microalgae consortia to sulfamethoxazole (SMX). We found that the removal efficiency of SMX by *C. pyrenoidosa* reached 49%, while the other four microalgae ranged between 9% and 16%. In addition, *C. pyrenoidosa*, S. *quadricauda*, and *Dictyosphaerium* sp. had better tolerance to SMX than *H. pluvialis*, and their growth and photosynthesis were less affected. At 10 and 50 mg/L SMX, the removal capacity of SMX by mixed microalgae consortia was lower than that of *C. pyrenoidos* except for the consortium with *C. pyrenoidos* and *S. quadricauda*. The consortia generally showed higher sensitivity towards SMX than the individual species, and the biochemical characteristics (photosynthetic pigment, chlorophyll fluorescence parameters, superoxide anion (O_2_^-^), superoxide dismutase activity (SOD), malondialdehyde (MDA) and extracellular enzymes) were significantly influenced by SMX stress. Therefore, the removal of antibiotics by microalgae consortia did not increase with the number of microalgae species. Our study provides a new perspective for the selection of microalgal consortia to degrade antibiotics.

## 1 Introduction

The relationship between biodiversity and ecosystem functioning is a subject of widespread concern and has been studied in both terrestrial and aquatic ecosystems (Diaz and Cabido, 2001). Increased species diversity typically leads to higher functional differences in the community, which may result in more effective access to and use of resources in turn (Loreau and Hector, 2001; Fargione et al., 2007). Several studies have shown that high species diversity of plants, animals, and microorganisms can resist the negative effects of a changing external environment (Ghorbannezhad et al., 2018; Norberg, 2000; Vacek et al., 2021). In addition, species diversity may have effect on the degradation of pollutants in the environment. For example, inoculating microbial communities isolated from *in-situ* contaminated soil can effectively improve the degradation of polycyclic aromatic hydrocarbons (Li et al., 2008). And microalgae consortia have proven to significantly improve the degradation of cefradine, p-chlorophenol, and other pollutants in the water environment in comparison with monocultures (Lima et al., 2004; Xiao et al., 2021). However, Choudhury et al. (2018) and Brisson et al. (2020) pointed out that mixed cultivation of plants is not necessarily better than single cultivation in resource utilization and pollutant removal.

Antibiotics, widely used in aquaculture andanimal husbandry, have become an important class of contaminants of emerging concern (Kümmerer, 2009; Liu et al., 2018; Li et al., 2020). Previous studies have shown that the removal of antibiotics depends on physical adsorption, advanced oxidation technology and microbial degradation (Dutta and Mala, 2020; Huang et al., 2021). Recently, microalgae-mediated biodegradation has attracted attention and proven to be an environmentally effective and safe antibiotic removal technology (Xiong et al., 2021; Yu et al., 2022). Microalgae such as *Chlorella* sp., *Dictyostelium* sp., and *Scenedesmus obliquus* have been shown to effectively degrade antibiotics (Chen et al., 2020a; Chen et al., 2020b; Yang et al., 2020; Aydin et al., 2022). Nevertheless, microalgae have specific preferences for antibiotic types, and different microalgae have different cell hydrophobicity, which affect the degradation of antibiotics (Bai and Acharya, 2016; Xiong et al., 2017c). Therefore, recent studies have focused on the consortia of microalgae and bacteria or other microalgae to improve the removal efficiency of antibiotics (Rodrigues et al., 2020; Rodrigues et al., 2021; Wang et al., 2022). However, whether the determinant of antibiotic removal efficiency of microalgae consortia is higher than for keystone species needs further studies.

We investigated the removal and tolerance capacity to sulfamethoxazole (SMX) of five green microalgae (*C. pyrenoidosa*, *S. quadricaudas*, *Dictyostelium* sp., *H. pluvialis* and *Botryococcus braunii*) and then screened several combinations of these microalgae for consortia effects. To explore the difference in the efficiency of removing antibiotics by different microalgae consortia, we measured a series of biochemical indicators. We hypothesized that the keystone microalgae species had a strong removal capacity for SMX. Besides, consortia of individual keystone microalgae with a high removal efficiency of SMX and other microalgae species may not enhance but potentially reduce the removal efficiency of SMX. Our results contribute to a better understanding of the ecotoxicity of SMX and its removal by microalgae and microalgae consortia.

## 2 Article types

Original Research.

## 3 Materials and methods

### 3.1 Chemicals

SMX was purchased from Sangon Biotech Co., Ltd (Shanghai, China) with >98% purity. We used 0.1% mol/L NaOH as a solvent to fully dissolve 0.1 g SMX. Then, we added 0.1 mol/L HCl to adjust the initial pH 7 of the test medium (Chen et al., 2020b). The chemicals were used of analytical grade.

### 3.2 Algal cultures

Five microalgae: *Chlorella pyrenoidosa* (FACHB-10), *Scenedesmus quadricauda* (FACHB-44), *Dictyosphaerium* sp. (FACHB-2072), *Haematoccocus pluvialis* (FACHB-1928) and *Botryococcus braunii* (FACHB-357) were purchased from the Freshwater Algae Culture Collection of the Institute of Hydrobiology (FACHB-Collection), Wuhan City, China. The microalgae were cultivated in 3000 mL Erlenmeyer flasks containing BG-11 medium and incubated in a homoeothermic incubator at 25 ± 1°C under 3000 lux illumination with a light-dark period of 12:12 h. To maintain the logarithmic growth of the microalgae and reduce any effect caused by minor differences in photo irradiance, the flasks were arranged randomly and gently shaken three times a day (Wan et al., 2015). All experimental devices used for the algal culture/for algal cultivation were autoclaved at 121°C for 30 min before use.

### 3.3 Experimental design

To explore differences in toxicity to and removal efficiency of antibiotics by keystone microalgae species in monocultures or when combined, we designed two experiments: microalgae screening and microalgae consortia.

#### 3.3.1 Microalgae screening

According to previous research and our pre-experiment results our preliminary experimental results on nine microalgae species (**Supplementary Figure 1**), we selected five microalgae, *C. pyrenoidosa*, *S. quadricauda*, *Dictyosphaerium* sp., *H. pluvialis* and *B. braunii*, which have shown tolerance and removal capacity to organic pollutants in the environment (Cheirsilp et al., 2016; Chen et al., 2020a; Yang et al., 2020; Aydin et al., 2022), as experimental objects and explored their tolerance to and removal capacity of SMX. The SMX solution was prepared with BG-11 medium with the experimental concentration of 5 mg/L and added to the five microalgae cultures. The experiment was then conducted in 500-mL Erlenmeyer flasks containing 400 mL of BG-11 medium inoculated with 15 mg/L microalgal cell suspension. Besides, the control comprised an algal medium without added SMX, and an abiotic control group contained only SMX ([CK]). Three replicates were set up for each treatment and control group, and all flasks were incubated under the same conditions used for the inoculum culture for 16 days. Samples were collected on days 2, 4, 6, 8, 10, 12, 14, and 16 to determine each index.

#### 3.3.2 Microalgae consortia

Based on the results of our first experiment, we selected three of the five species: *C. pyrenoidosa*, *S. quadricauda* and *Dictyosphaerium* sp. *C. pyrenoidosa*, which had the strongest tolerance and removal capacity to SMX, was selected as control (C), and groups where *C. pyrenoidosa* was combined with the other two microalgae respectively acted as treatments (**Table 1**). All microalgae in the treatment group were inoculated at the same fresh weight ratio, and the total content of microalgae in the final system was 15mg/L. Microalgae consortia experiments were carried out with 10 and 50 mg/L SMX, respectively. According to the microalgae screening experimental results, SMX can be mostly removed by microalgae in the first 7 days. Therefore, a 7-days exposure experiment was conducted where photosynthetic pigments, photosynthesis, and the activities of intracellular and extracellular enzymes of microalgae consortia were measured.

**Table 1:** Experimental setup of the combined experiment.

Group name	Microalgae species
CK	Abiotic control group
C	*C. pyrenoidosa*
C+S	*C. pyrenoidosa* + *S. quadricauda*
C+D	*C. pyrenoidosa* + *Dictyosphaerium* sp.
C+S+D	*C. pyrenoidosa* + *S. quadricauda* + *Dictyosphaerium* sp.

### 3.4 Measurement of cell growth

Algal cell growth in each treatment was measured according to Peng et al. (2014). The algal biomass of five microalgae was calculated according to its linear relationship with an optical density of 680nm (OD_680_) (**Supplementary Table 1**).

### 3.5 Measurement of photosynthetic pigment and chlorophyll fluorescence parameters

The modified hot methanol method was used to determine the cytochrome of microalgae (Xiong et al., 2016). Briefly, 5 mL of the sample was centrifuged at 6500 rpm for 15 min. After removing the supernatant, 5 mL of 95% methanol was added to the sample, which was then placed in a 60 °C constant temperature water bath for 10 min. The mixed samples were centrifuged at 6500 rpm for 15 min, and absorbance of the supernatant at 665, 652, and 470 nm was measured with a spectrophotometer. Chlorophyll a, chlorophyll b and carotenoids were calculated as follows:


$$Chlorophyll\;a\;\left(Ca,\;mg/L\right)\;=\;16.82\;A_{665}-9.28\;A_{652}$$

$$Chlorophyll\;b\;\left(Cb,\;mg/L\right)\;=\;36.92\;A_{652}-16.54\;A_{665}$$

$$Carotenoids\;\left(mg/L\right)\;=\;\left(1000\;A_{470}-1.\;91Ca\;-\;95.\;15Cb\right)/225$$

Moreover, chlorophyll fluorescence parameters, including photosynthetic system II (PSII) maximum photosynthetic rate (*Fv/Fm*) and actual photosynthetic efficiency (*Yield*), were measured using a pulse amplitude-modulated fluorometer (Phyto-PAM, Walz, Effeltrich, Germany) equipped with an emitter-detector-fiberoptic unit with an irradiance of 16 *μ*mol photons/m^2^/sec PAR.

### 3.6 Antioxidant responses

Samples of microalgae suspension (5 mL) were collected on day 3 and 7 to determine superoxide anion. The samples were centrifuged at 7000 rpm for 20 min, and then the supernatant was removed. Then 1 ml of extract was added, crushed with a cell crusher for 18 min, and centrifuged at 10000 rpm at 4 °C for 20 min. The concentration of O_2_^-^ was determined according to the assay kit (Beijing Solabao Technology Co., Ltd., China).

In addition, the concentrations of SOD and MDA were determined using assay kits (Nanjing Jiancheng Bioengineering Institute, China) following the manufacturer’s protocol. The extraction steps were as follows: 1) 10 mL samples were collected and centrifuged at 6500 rpm for 10 min at 4°C (Eppendorf, Centrifuge 5810R), 2) the biomass pellet was washed with 0.01 M sodium phosphate buffer (pH 7.4), crushed and centrifuged, and 3) the enzymes obtained from the disrupted microalgae were extracted using 500μL of sodium phosphate buffer.

### 3.7 Determination of extracellular enzyme content

The change characteristics of extracellular enzyme activity of microalgae were determined by API ZYM (Merier, France), which is a laboratory kit for semi-quantitative analysis of the production of hydrolytic enzymes. 3mL samples were collected and centrifuged at 7000 rpm for 15 min to obtain the supernatant containing extracellular enzymes. Supernatants (65 μL) were dispensed into the 20 microcupules of API ZYM strips and incubated at 37°C for 4 h. After that, 30 μL of the commercial reagents ZYM A and ZYM B was added to all the microcupules to develop chromogenic substrates. The color reactions were read after 5 min, and the results were recorded at four grades from 0 to 4 according to the color rendering degree in the microcupules (Martinez et al., 2016).

### 3.8 Determination of antibiotic concentration

The samples were filtered through a 0.22 μm polytetrafluoroethylene (PTFE) filter, and the residual SMX concentration was determined by high performance liquid chromatography (HPLC, Alliance 2695 system, USA). A total of 2 μL of the samples was separated using an ACQUITY UPLC BEH C18 chromatography Column [250 × 4.6 mm, 5 µm, waters, USA) at a column temperature of 30°C. The mobile phase consisted of a mixture of solution A (acetonitrile and LCMS-grade water (30: 70 v/v)] and solution B (0.1% formic acid in LCMS-grade water) at a flow rate of 1.0 mL/min.

The removal rate (%) of SMX was calculated using the following formula:


$$Removal\;rate\;\left(\%\right)\;=\;\left(1-C_{t}/C_{0}\right)\;\times \;100\%$$

where *C_0_* (mg/L) is the initial concentration of antibiotics at time *0*, and C*_t_* (mg/L) is the concentration of antibiotics at time *t*.

### 3.9 Statistical analysis

To determine significant differences between the control and treatment groups, a one-way analysis of variance (ANOVA) was conducted using SPSS statistics software (version 22, Chicago, IL, USA). Values with *p*< 0.05 and *p*< 0.01 were considered significant and very significant, respectively. To reveal the relationship between the variation of various indicators of microalgae and the experimental period, principal component analysis (PCA) was applied using R 4.1.1 statistical computation software. In addition, hierarchy cluster analysis of the extracellular enzyme was performed using “pheatmap” package in R 4.1.1 statistical computation software. All figures were generated using Origin 2021 software (OriginLab, Northampton, MA, USA).

## 4 Results

### 4.1 Microalgal screening

#### 4.1.1 Effects of SMX on algal growth and photosynthesis

**Figure 1:**
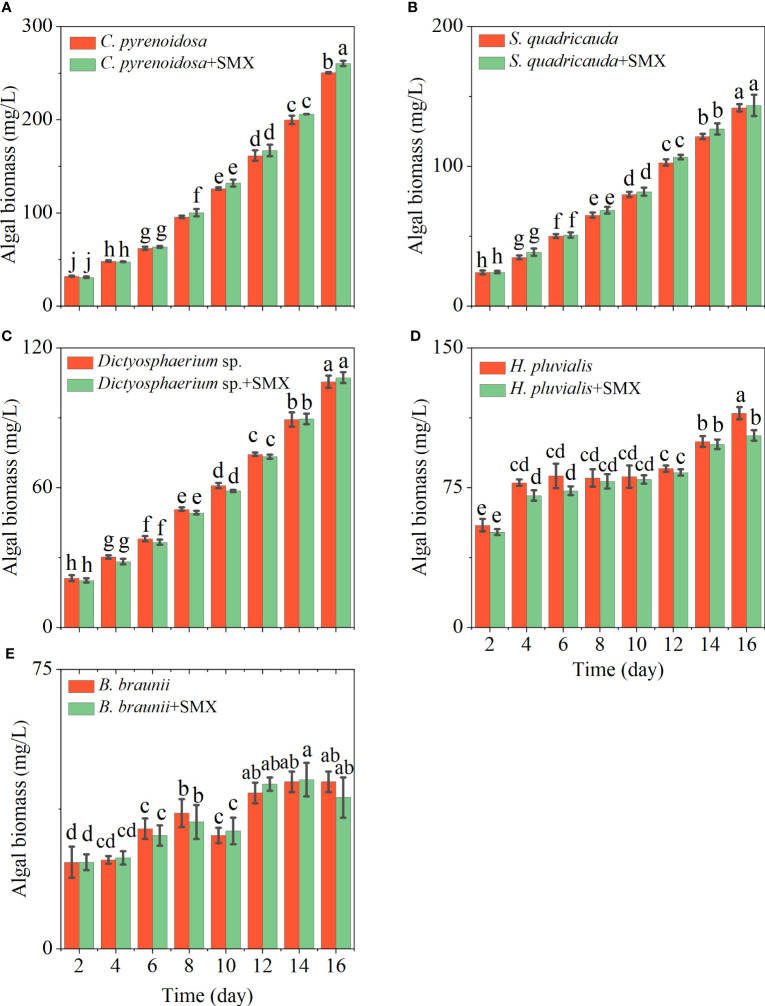
Differences in the algal biomass of five microalgae (**A**: *C. pyrenoidosa*, **B**: *S. quadricauda*, **C**: *Dictyosphaerium* sp., **D**: *H pluvialis* and **E**: *B braunii*) during 16 days of cultivation. Error bars represent standard deviation (n=3). Columns with different letters indicate significant differences (*p*<0.05) between the control and treatment.

The growth ability of the five microalgae differed, and they also had different responses to SMX (**Figure 1**). Compared with the control groups, *C. pyrenoidosa*, *S. quadricauda* and *Dictyostelium* sp. grew better in the medium containing SMX, especially the biomass of *C. pyrenoidosa* increased significantly compared with the control group (*p*<0.05) (**Figures 1A, C, D**). By contrast, the growth of *H. pluvialis* and *B. braunaii* was significantly inhibited (*p*<0.05) (**Figures 1B, E**). During the course of the experiment, the *μ* of five microalgae showed a decline to a varying degree, most pronounced for *H. pluvialis*.

**Figure 2:**
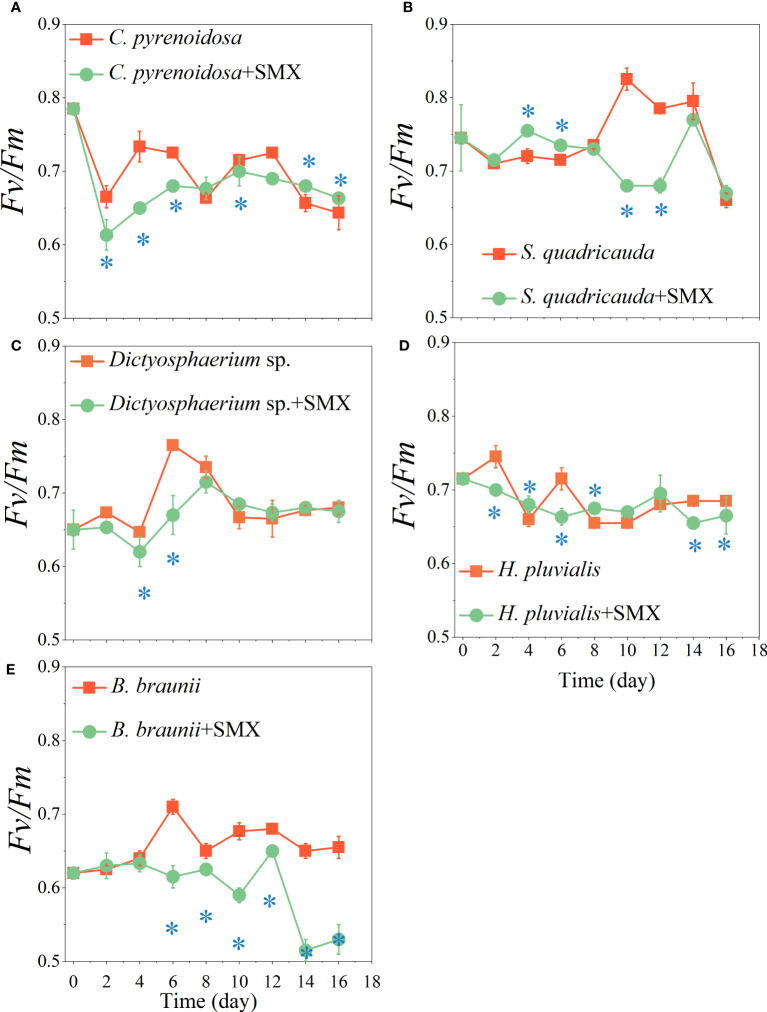
Effects of the SMX on the maximum photosynthetic efficiency (Fv/Fm) of five microalgae (**A**: *C. pyrenoidosa*, **B**: *S. quadricauda*, **C**: *Dictyosphaerium* sp., **D**: *H pluvialis* and **E**: *B braunii*) during 16 days of cultivation. Error bars represent standard deviation (n=3). Asterisks indicate significant differences between the control and treatment groups (*p*< 0.05*).

After exposure to SMX, *F_v_/F_m_* of the microalgae was significantly reduced (*p*<0.05) except for *S. quadricauda* early in the experiment (**Figure 2**). On day 14 and 16, *F_v_/F_m_* of *C. pyrenoidosa* exposed to SMX was significantly higher than that of the control group (*p*<0.05) (**Figure 2A**), *F_v_/F_m_* of *Dictyostelium* sp. and *S. quadricauda* were not higher (**Figures 2B, C**), for *H. pluvialis* and *B. braunaii* it was significantly lower (*p*<0.05) (**Figures 2D, E**). SMX did not exert obvious stress on *C. pyrenoidosa*, *S. quadricauda*, and *Dictyostelium* sp. but promoted the synthesis of chlorophyll a (**Supplementary Figure 2**). In addition, during the whole experimental period, the *Yield* of the microalgae treatment group was different from that of the control group, especially that of the *B. braunaii* treatment group showed a very significant decrease (*p*<0.01) (**Supplementary Figure 3**).

#### 4.1.2 SMX removal of five microalgae

**Figure 3:**
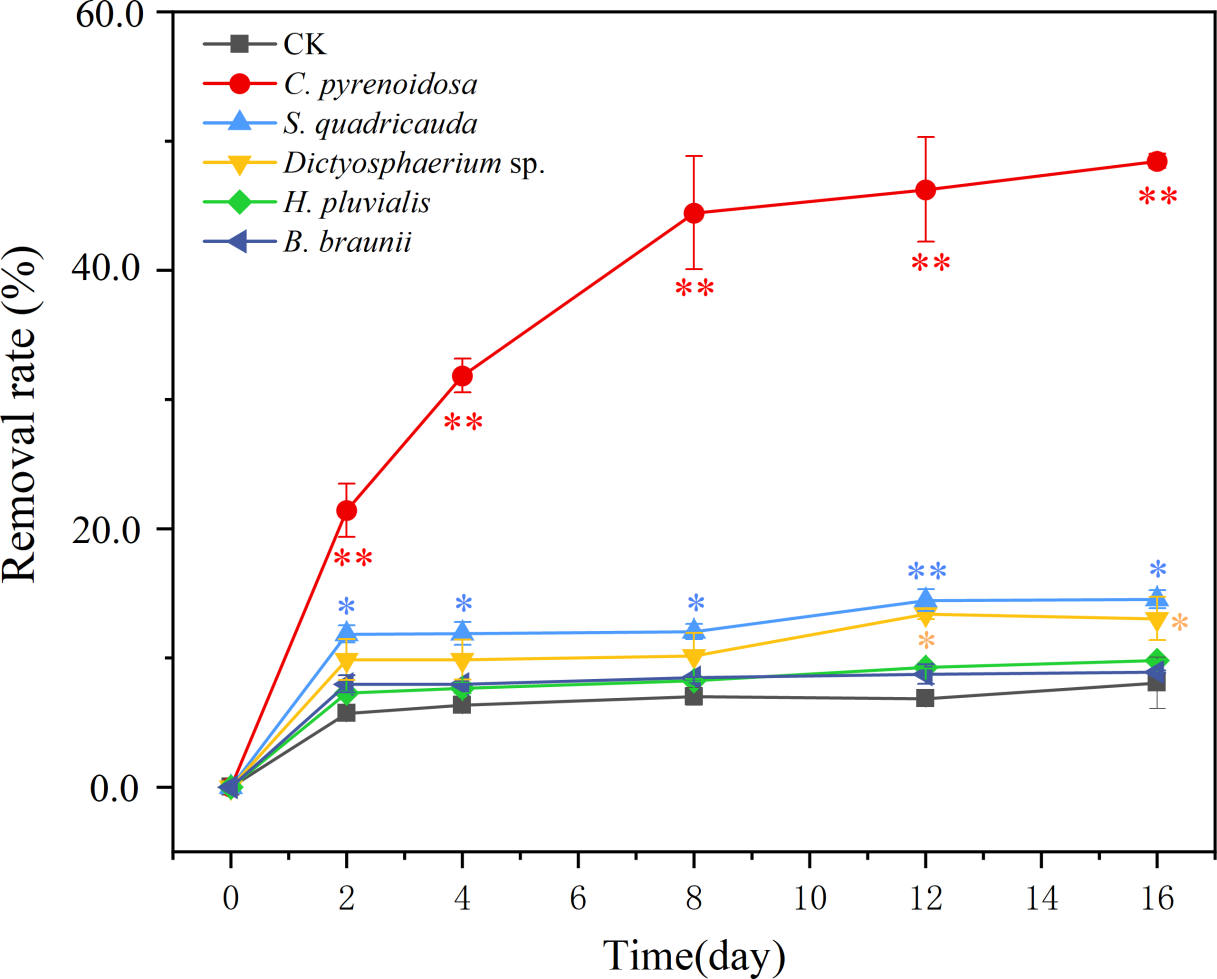
The removal rate (%) of SMX for five microalgae during 16 days of exposure. CK was an abiotic control containing 5 mg/L SMX. Error bars represent standard deviation (n=3). Asterisks indicate significant differences between the control and treatment groups (*p*< 0.05*; *p*< 0.01**).

*C. pyrenoidosa* had the highest removal rate of SMX (49%) during the 16 days exposure. The removal rates of *S. quadricauda* and *Dictyostelium* sp. were 16% and 13%, respectively, and 10% and 9% for *H. pluvialis* and *B. braunii*, respectively. (**Figure 3**). Compared with CK group, the removal rate of SMX by *C. pyrenoidosa* was significantly increased (*p*<0.01), and higher than that of the other four microalgae. Besides, the removal rate of SMX by *S. quadricauda* and *Dictyostelium* sp. was also significantly higher than that of CK group (*p*<0.05).

### 4.2 Microalgae consortia

#### 4.2.1 Effects of SMX on pigment and photosynthetic parameters

**Figure 4:**
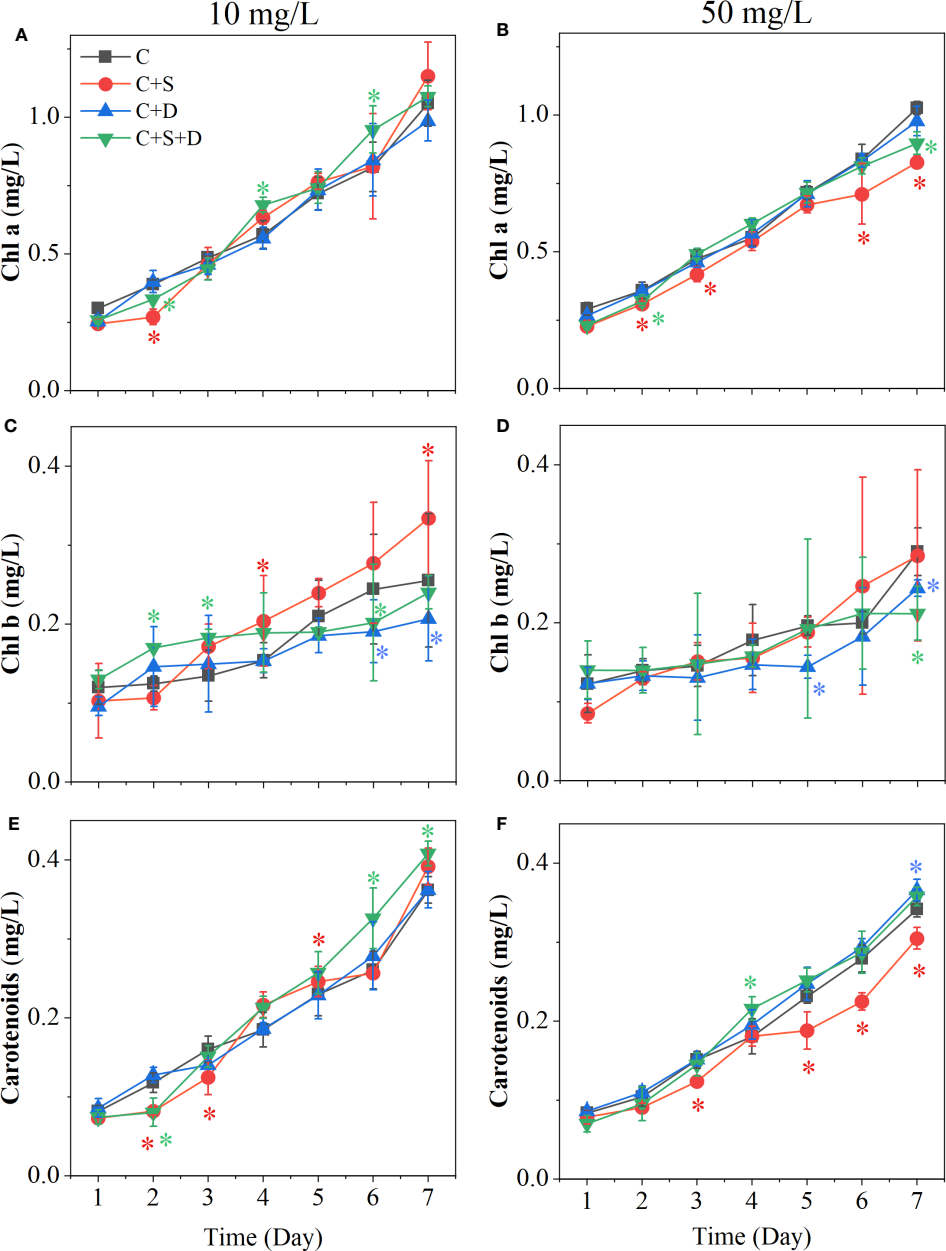
Effects of two concentrations of SMX on the cytochromes of different microalgae consortia during 7 days. **(A, C, E)** 10 mg/L; **(B, D, F)** 50 mg/L. Error bars represent standard deviation (n=3). Asterisks indicate significant differences between the control and treatment groups (*p*< 0.05*).

At different concentrations of SMX treatment, the content of three photosynthetic pigments in each treatment group showed upward trends (**Figure 4**). However, when the concentration of SMX increased to 50 mg/L, the total amount of photosynthetic pigment synthesis decreased (**Figure 4**). When treated with 10 mg/L SMX, there was no significant difference in chlorophyll a content between the treatment and control groups at the end of the experiment. However, when SMX reached 50 mg/L, the chlorophyll a content of [C+S] group and [C+S+D] group was significantly lower than in the control group (*p*<0.05) (**Figures 4A, B**). at the two SMX concentrations, the content of chlorophyll b in [C+S] group fluctuated greatly, but the content of chlorophyll b in [C+D] group and [C+S+D] group was significantly lower than in the control group (*p*< 0.05) (**Figures 4C, D**). In addition, the change of carotenoids in the [C+S] group fluctuated more at the two SMX concentrations. (**Figures 4E, F**). When exposed to SMX on day 2, *Fv/Fm* of all groups was significantly inhibited (*p*<0.05) and then gradually recovered on day 3 (**Supplementary Figures 4A, B**). *Yield* was significantly higher for all groups than in the control group (*p*<0.05) (**Supplementary Figures 4C, D**).

#### 4.2.2 Effect of SMX on the intracellular antioxidant system of microalgae

**Figure 5:**
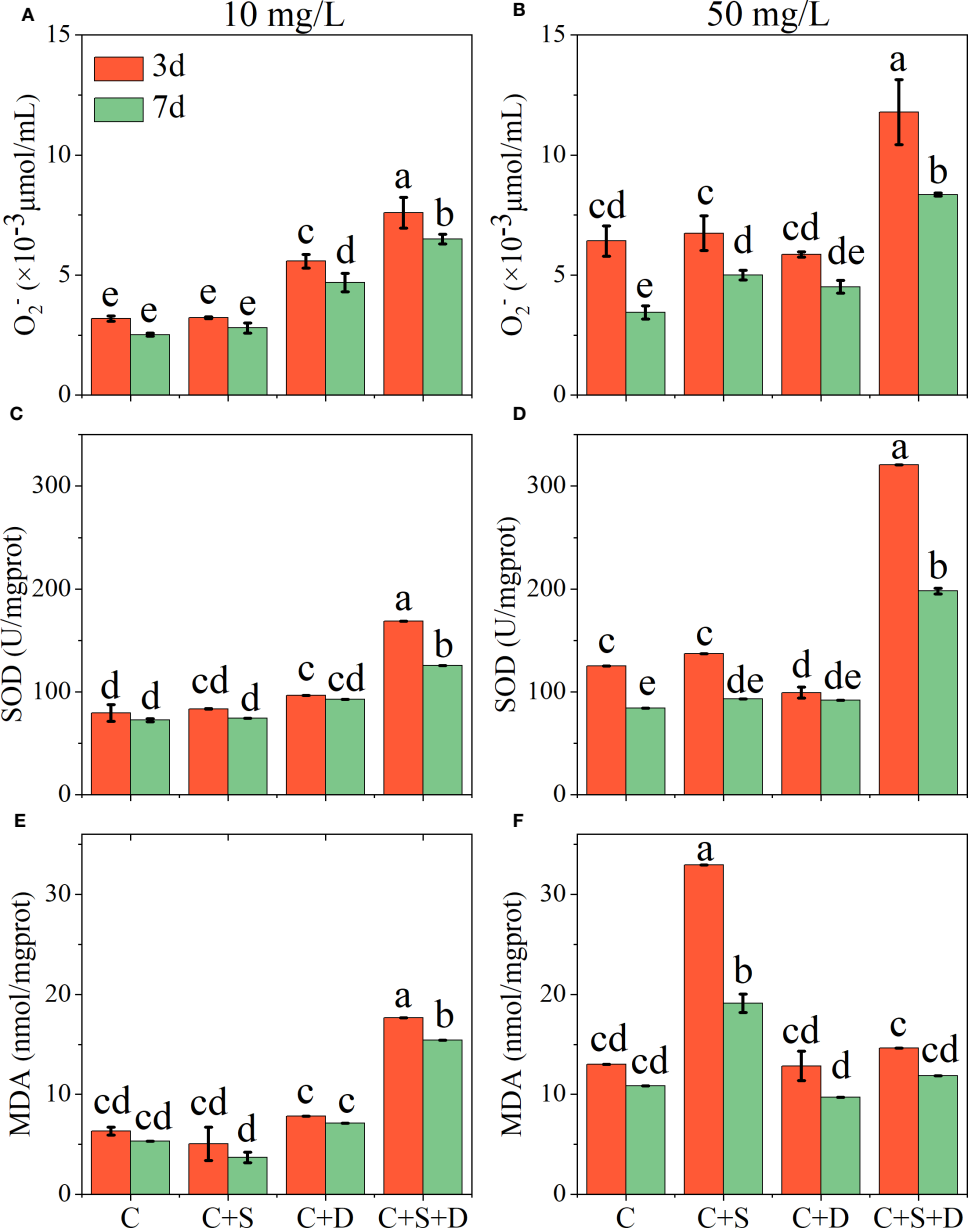
Effects of different concentrations of SMX on the intracellular antioxidant system of microalgae consortia during 7 days of exposure. Superoxide anion (O_2_^-^) content **(A, B)** superoxide dismutase (SOD) activity **(C, D)** malondialdehyde (MDA) content **(E, F)**. Error bars represent standard deviation (n=3). Columns with different letters indicate significant differences (*p<*0.05) between the control and treatment groups.

In the 10 mg/L SMX treatment, the concentrations of O_2_^-^, SOD, and MDA in the [C] group, [C+S] group, and [C+D] group showed no significant change on day 3 and day 7 (**Figures 5A, C, E**). However, the contents of O_2_^-^, SOD, and MDA in [C+S+D] group were significantly higher than in the other groups, and the content on day 3 was significantly higher than on day 7 (*p*<0.05). When SMX reached 50 mg/L, the contents of O_2_^-^ and SOD in the [C+S+D] group increased significantly compared with those in the 10 mg/L SMX treatment, and the contents on day 3 were 1-fold and 1.6-fold higher than on day 7, respectively. In addition, the contents of O_2_^-^ and SOD in [C] group and [C+S] group were significantly higher on day 3 than on day 7 (*p*<0.05), but there was no significant change in [C+D] group (**Figures 5B, D**). However, the content of MDA in [C+S] group was significantly higher than in the other groups below 50 mg/L SMX treatment (*p*<0.05), and the content on day 3 was 1.7-fold higher than on day 7. The content of MDA in [C+S+D] group was not significantly different from that in the 10 mg/L SMX treatment, and the MDA content exhibited no significant change on day 3 and day 7 as the [C] group and the [C+D] group (**Figure 5F**).

#### 4.2.3 Effects of SMX on extracellular enzyme

**Figure 6:**
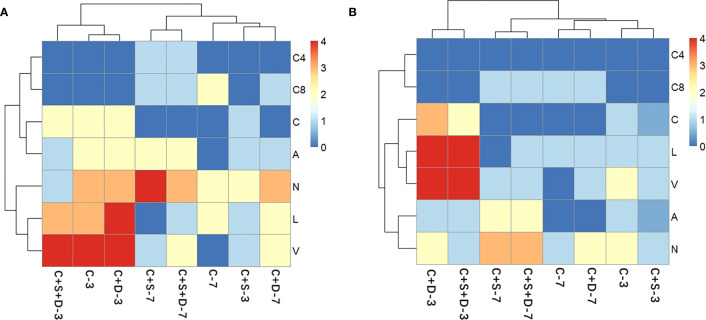
Heat map analysis of the extracellular enzyme of different microalgae consortia on day 3 and day 7 of exposure to SMX at three concentrations: 10 mg/L **(A)** and 50 mg/L **(B)**. The names of the different types of extracellular enzymes are distinguished by letters (C4, esterase; C8, lipase esterase; C, Cystine aminopeptidase; A, Acid phosphatase; N, Naphthyl-AS-BI-phosphate; L, Leucine aminopeptidase; V, Valine aminopeptidase). The color of each section is proportional to the significance of change in the biochemical parameters (red, relatively high; blue, relatively low).

The heatmap revealed changes in the activity of extracellular enzymes throughout the experiment. Overall, 7 of the 20 enzymes could be detected on day 3 and day 7 of the experiment, respectively, namely acid phosphatase, naphthol-AS-BI-phosphate, esterase (C4), lipase esterase (C8), leucine aminopeptidase, valine aminopeptidase and cystine aminopeptidase (**Figure 6**). At both SMX concentrations, C4 and C8 did not appear on day 3 of the experiment but were recorded on day 7, and the number and activity decreased with the increasing SMX concentration. The activities of the three aminopeptidases were greater on day 3 than on day 7, being higher in the [C+D] and [C+S+D] groups and lower in the [C+S] group. In addition, with the increased SMX concentration, the activity of phosphatase gradually decreased in all treatment groups but the activity on day 7 of the experiment was higher than that on day 3.

#### 4.2.4 Removal of SMX by different microalgae consortia

**Figure 7:**
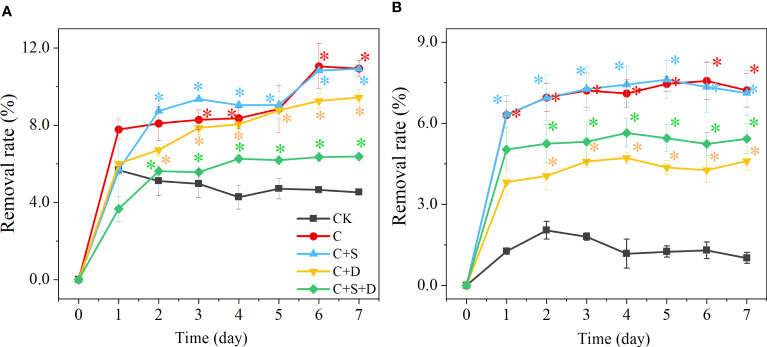
The removal rate (%) at different concentrations of SMX in three different microalgae consortia treatment groups **(A)** 10 mg/L; **(B)** 50 mg/L. Error bars represent standard deviation (n=3). Asterisks indicate significant differences between the control and treatment groups (*p*< 0.05*; *p*< 0.01**).

The antibiotic content of treatment groups decreased significantly during the first two days of the experiment (*p*<0.05). With enhanced SMX concentration, the removal capacity of all treatment groups decreased. [C] group and [C+S] group had a stronger removal capacity than the other groups whose removal rates reached 11.1% and 10.9%, respectively, at 10 mg/L SMX. However, although [C+D] and [C+S+D] had the capacity to remove the different concentrations of SMX, SMX was significantly lower than for the [C] and [C+S] groups (*p*<0.05) (**Figure 7**).

#### 4.2.5 PCA

**Figure 8:**
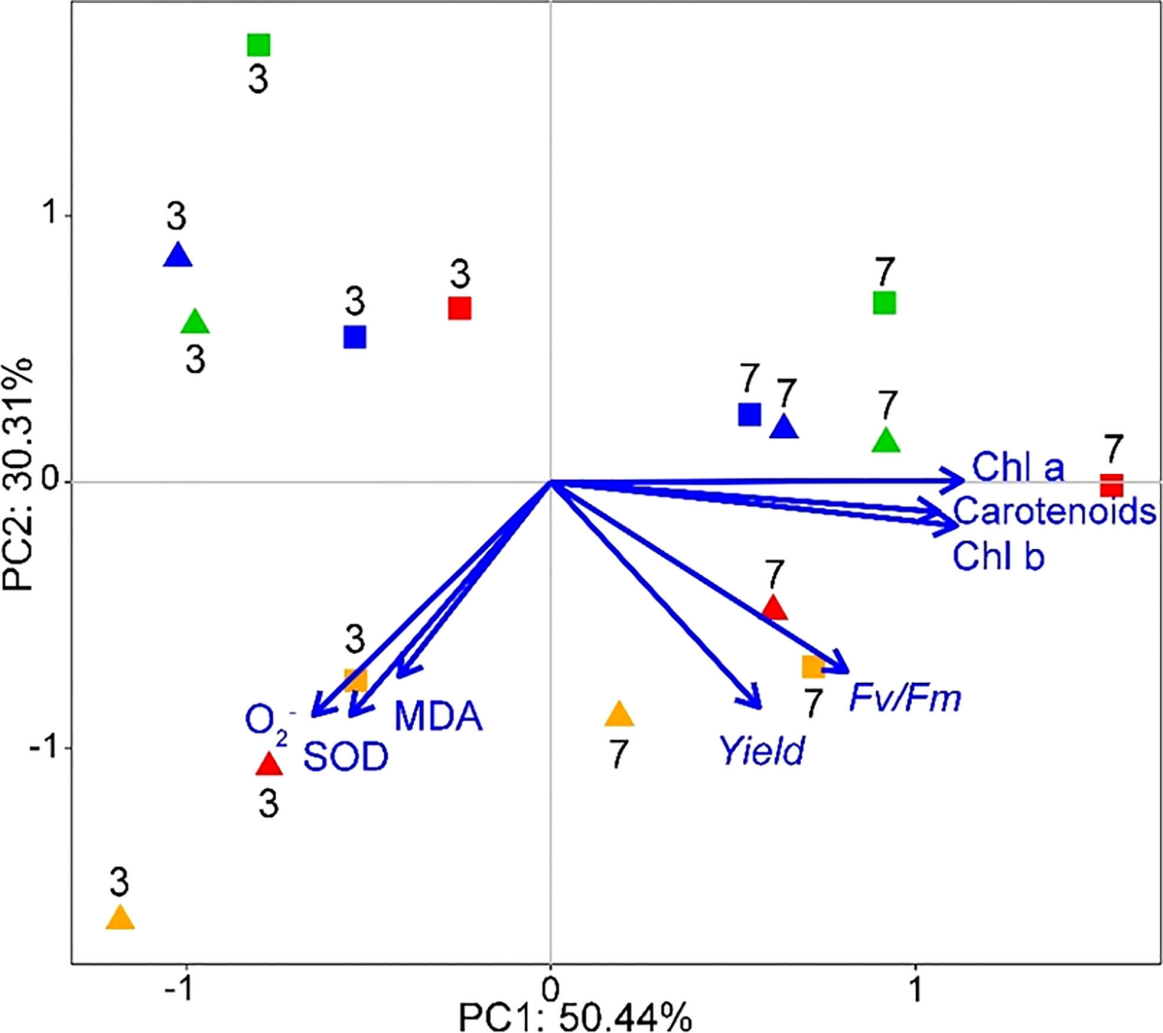
Biplot from PCA integrating all the measured variables (Chl a, Chl b, carotenoids, *F_v_/F_m_*, *Yield*, MDA, O_2_^-^, SOD) for the two sampling times (days 3 and 7) and eight different treatments (shapes and colors interact in pairs: ◼-10 mg/L, ▲-50 mg/L; green-[C] group, red-[C+S] group, blue-[C+D] group, orange-[C+S+D] group).

On day 3 and 7 of the SMX treatment, PC1 constituted 50.4% of the total variance in all groups, and PC2 accounted for an additional 30.3% (**Figure 8**). The distribution of each microalgae consortium in PCA was significantly different on day 3 and day 7 after exposure to SMX. On day 3, the contents of O_2_^-^, SOD, and MDA of the microalgae consortia had changed most significantly, while on day 7 mainly photosynthetic pigment and chlorophyll fluorescence parameters of microalgae consortia had changed.

## 5 Discussion

We found that the tolerance and removal efficiency of SMX by the five different microalgae varied. *C. pyrenoidosa* showed the highest removal capacity, followed by *S. quadricauda* and *Dictyosphaerium* sp. Previous studies have shown that *Chlorella* sp. has excellent dissipation to many antibiotics, so it is often the preferred microalgae to remove antibiotics (Song et al., 2019; Chen et al., 2020b; Xiong et al., 2020). Besides, *C. pyrenoidosa* had better growth and higher photosynthesis in the culture system containing SMX. Other studies have shown increases in the biomass of *C. pyrenoidosa* treated with both chloramphenicol and florfenicol (Lai et al., 2009). Stimulated growth might reflect that *C. pyrenoidosa* can resist the persecution of antibiotics at low concentrations and even decompose and absorb antibiotics as organic substances (Kiki et al., 2020). Some studies have shown that *Dictyosphaerium* sp. can combine with the photocatalyst bismuth vanadate (BiVO_4_) to improve the removal of sulfamethazine (SM2), while *H. pluvialis* can efficiently degrade nearly 10 kinds of antibiotics through membrane photobioreactors (Chen et al., 2020c; Claude et al., 2022). Due to the different modes of action and drug affinity of microalgae, these microalgae did not show high degradation rates for SMX in this study. Therefore, *C. Pyrenoidosa* is a keystone microalgae species for degrading SMX. However, when we combined *C. pyrenoidosa* with two other microalgae, the microalgae consortia showed a higher photosynthetic pigment content and stronger photosynthesis than in the *C. pyrenoidosa* monoculture after the SMX treatment, and the activity of O_2_^-^ and antioxidant enzymes in the antioxidant system of the microalgae consortia was higher than for the individual microalgae. The PCA results also revealed that the effects of SMX on antioxidant enzymes and photosynthesis of microalgae consortia were stronger than that of individual microalgae species *C. pyrenoidosa*. Chlorophyll is an important component of light capture and energy transduction in photosynthesis and photosynthetic reaction, including light reaction, Calvin cycle, and starch synthesis. Some emerging pollutants can affect the accumulation of chlorophyll content in microalgae (Telfer, 2002; Del Campo et al., 2007). And the increase in chlorophyll content in cells can serve as a protective mechanism to eliminate the accumulation of reactive oxygen species in chloroplasts (Kasahara et al., 2002; Tsiaka et al., 2013; Xiong et al., 2016). In addition, the antioxidant properties of carotenoids can prevent lipid peroxidation by inhibiting the production of singlet oxygen and free radicals in cells, thereby promoting the stability of photosynthesis and protecting cells (Jahns and Holzwarth, 2012). When the antioxidant defense system is stimulated under stress conditions, its antioxidant enzyme activity will increase (Qian et al., 2008; Singh et al., 2018). Throughout the entire experimental cycle, the changes in photosynthetic pigments and antioxidant enzyme activity of microalgae consortia were significantly stronger than those of individual microalgae. Our results, therefore, indicate that the microalgae consortia were more sensitive to SMX than the individual microalgae species, which is consistent with the findings of Xiong et al. (2017b) in their study of the effects of enrofloxacin on microalgae consortia.

When *C. pyrenoidosa* was combined with other microalgae, the removal efficiency of SMX was never higher than that of the individual keystone microalgae *C. pyrenoidosa*, and it even declined for some of the consortia: the removal efficiency of [C+S] to SMX was close to that of keystone microalgae *C. pyrenoidosa*, but the removal capacity of [C+S+D] group was weaker than that of the keystone microalgae and the consortia containing two microalgae species ([C+S] and [C+D]). Thus, the increase in the richness of the different microalgae taxa did not improve the removal rate of SMX. Lima et al. (2003) found that the higher the proportion of the core microalgae *Coenochloris pyrenoidosa* in the microalgae consortia, the higher the degradation rate of the microalgae consortia to *p*-nitrophenol. Therefore, the lower removal rate of SMX in the [C+S+D] group may likely be due to the decreased proportion of the keystone microalgae *C. pyrenoidosa* in the consortia because of resource competition. Besides, In the study on the co- metabolism of SMX by *C. pyrenoidosa*, it was found that the addition of organic substrate or other nutrients can accelerate the potential of microbial biodegradation of antibiotics, which is related to the characteristics of additional substrates that can maintain biomass production and act as an electron donor for co-metabolism of non-growth substrates (Xiong et al., 2020; Gao et al., 2022). Therefore, we speculate that co-cultivation of multiple microalgae may lead to competition for organic matter in the substrate, thereby affecting the metabolism of SMX by the microalgae itself. In co-culture systems, there are interactions between microalgae species, such as competition or symbiosis, so their capacity to degrade pollutants may also be affected by such interactions (White et al., 2014; Goncalves et al., 2017). The reported mechanisms of antibiotic removal by microalgae include photolysis, biosorption, bioaccumulation, and intracellular and extracellular biodegradation (Liu et al., 2021; Yu et al., 2022). In our study, photodegradation to remove SMX was negligible, and other studies (Xiong et al., 2016; Xiong et al., 2017a) have revealed that the biosorption and bioaccumulation of emerging pollutants contribute little to microalgae-mediated bioremediation. Therefore, the main mechanism of SMX removal by microalgae is the biodegradation induced by the enzyme system of microalgae. The results of the API-ZYM reagent strip demonstrated that the extracellular enzyme activity of [C+S] group was always low even with the increase of SMX concentration, while the extracellular aminopeptidase and phosphatase of [C+D] group and [C+S+D] group were active. Nitrogen compounds are the products of the nitrogen cycle, which begins with the hydrolysis of protein by aminopeptidase (Mayer, 1989), and phosphatase participates in the phosphorus cycle (Martinez et al., 2016; Hu et al., 2022). The substances produced by these cycles can maintain the growth of microorganisms. Therefore, there may be a symbiotic relationship between *C. pyrenoidosa* and *S. quadricauda* (Johnson and Admassu, 2013; Koreiviene et al., 2014), which can maintain a stable growth state under SMX stress. The symbiotic relationship between them may have been destroyed when adding *Dictyosphaerium* sp., because they need to mobilize more extracellular enzymes to decompose and cycle nutrients. SMX can promote gene expression by affecting the metabolic process of non-coding RNA, leading to the destruction of the ultrastructure of microalgae cells, affecting the permeability of cell membranes and the activity of antioxidant enzymes (Xu et al., 2022). In this study, the intracellular antioxidant enzymes of the [C+S+D] group were always in a more active state, and the content of O_2_^-^ was always at a higher level. This shows that under the stress of SMX, the balance between the generation and elimination of O_2_^-^ was destroyed, and the antioxidant enzyme could not effectively remove excessive O_2_^-^, so the physiological function and growth of the [C+S+D] group were under more severe stress. Additionally, when the SMX reached 50 mg/L, the MDA content of [C+S] group significantly increased and exceeded that of the other microalgae consortia. MDA is an aldehyde product produced by lipid peroxidation caused by ROS, which reflects the degree of lipid peroxidation of cell membranes and the tolerance of plants to stress (Celekli et al., 2013). MDA can also be used as an indicator of NADPH-dependent oxidase and peroxidase, which can effectively transform the accumulated pollutants in cells through various enzyme systems (Apel and Hirt, 2013). Therefore, differently from the control group [C], the increase of MDA may be a way to improve the antibiotic removal efficiency in the [C+S] group.

## Data availability statement

The original contributions presented in the study are included in the article/**Supplementary Material**. Further inquiries can be directed to the corresponding author.

## Author contributions

RH: Experiment, Analysis, Visualisation, Writing draft, Writing-review and editing. WL: Experiment, Analysis, Visualisation, Writing-review and editing. JS: Supervision, Writing-review and editing. SL: Experiment, Analysis and Writing-review. LW: Supervision, Project administration. EJ: Writing-review and editing. WZ: Supervision, Project administration, Writing-review and editing. All authors contributed to the article and approved the submitted version.
