# Supplementary material for: Keystone microalgae species determine the removal efficiency of sulfamethoxazole: a case study of Chlorella pyrenoidosa and microalgae consortia
Source: Front Plant Sci. 2023 Jul 5;14:1193668. doi: 10.3389/fpls.2023.1193668 (PMC10354436; doi:10.3389/fpls.2023.1193668)
Supplement: Supplementary file 1 [file DataSheet_1.docx]

Supplementary Material

Keystone microalgae species determine the removal efficiency of sulfamethoxazole: a case study of *Chlorella pyrenoidosa* and microalgae consortia

Ruohan Huang^1 #^, Wan Liu^1,3 #^, Jinghua Su^2^, Shihao Li^1,3^, Liqing Wang^1^, Erik Jeppesen^4, 5, 6,7^, Wei Zhang^1*^

*** Correspondence:** Wei Zhang: [weizhang@shou.edu.cn](mailto:weizhang@shou.edu.cn)

## Supplementary Tables

Supplementary Table 1. Linear relationships between algal biomass and OD680

| Algae | Algal biomass (mg/L) |
| --- | --- |
| *C. pyrenoidosa* | 510.63x+0.3207（R^2^=0.9998） |
| *S. quadricauda* | 903.08x+1.6675（R^2^=0.9993） |
| *Dictyosphaerium* sp. | 759.39x-2.4241（R^2^=0.9996） |
| *H. pluvialis* | 1086.9x+2.9776（R^2^=0.9990） |
| *B. braunii* | 1797.6x+1.0536（R^2^=0.9995） |

## Supplementary Figures


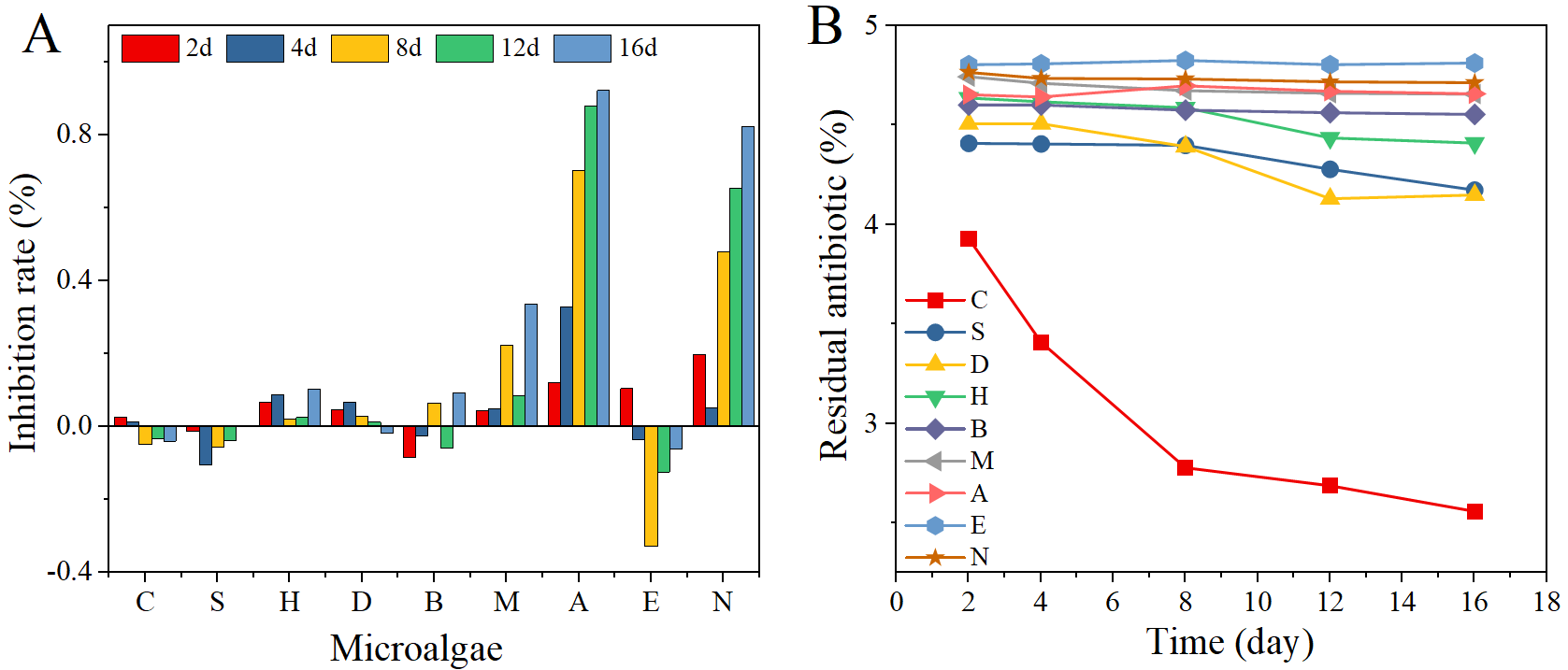


**Supplementary Figure 1.** A: growth inhibition rate of nine microalgae under 5 mg/L SMX treatment; B: the residual antibiotic of SMX of nine microalgae during 16 days. (C: *C. pyrenoidosa*; S: *S. quadricauda*; H: *H. pluvialis*; D: *Dictyosphaerium* sp.; B: *B. braunii*; M: *Mougeotia* sp.; A: *Aphanizomenon flosaquae*; E: *Euglena gracilis*; N: *Nitzschia* sp.)


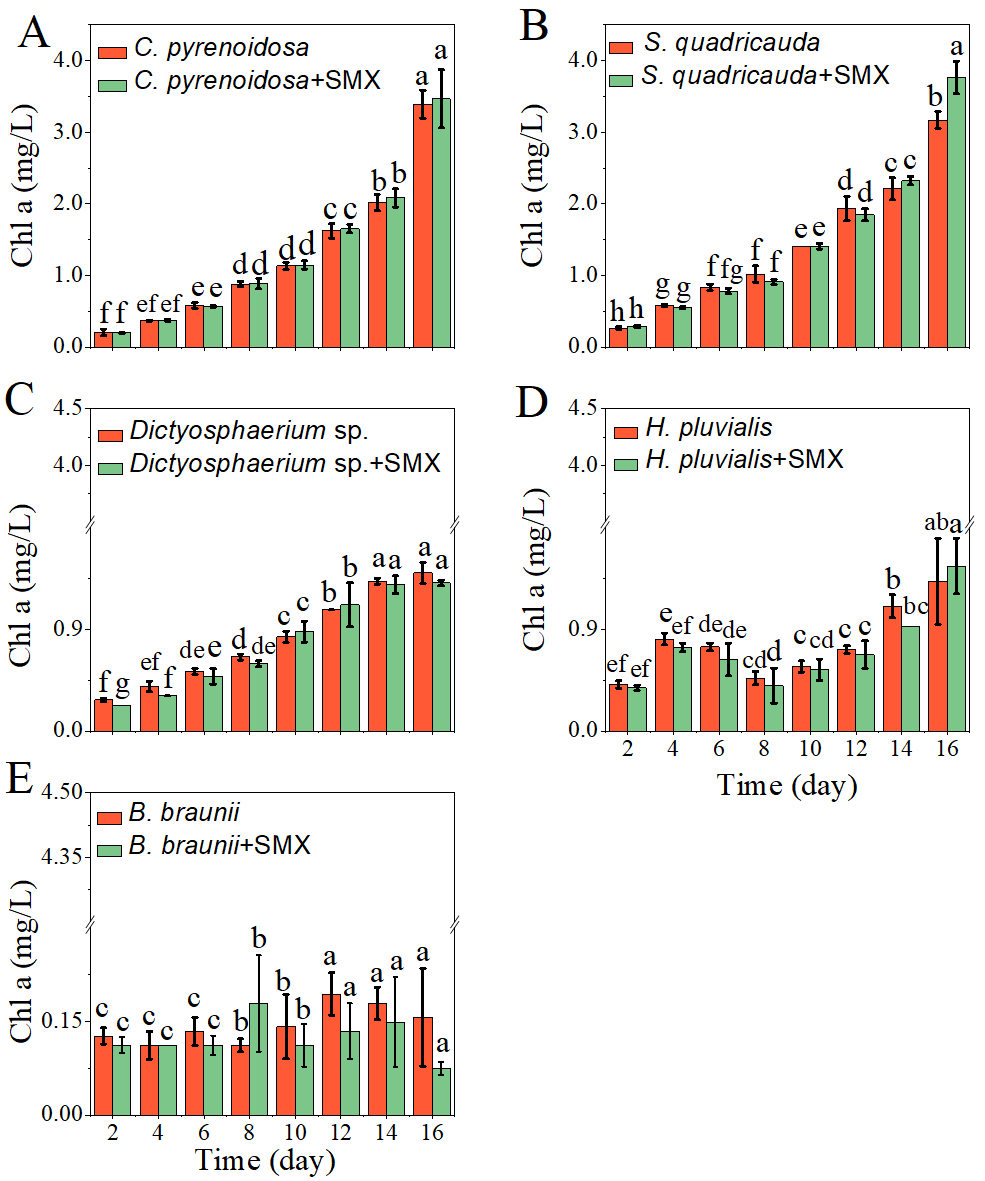


**Supplementary Figure 2.** Effects of SMX on chlorophyll a of five microalgae (*C. pyrenoidosa*, *S. quadricauda*, *Dictyosphaerium* sp., *H. pluvialis* and *B. braunii*) during 16 days of cultivation. Error bars represent standard deviation (n=3). Columns with different letters indicate significant differences (*p* <0.05) between the control and treatment groups.


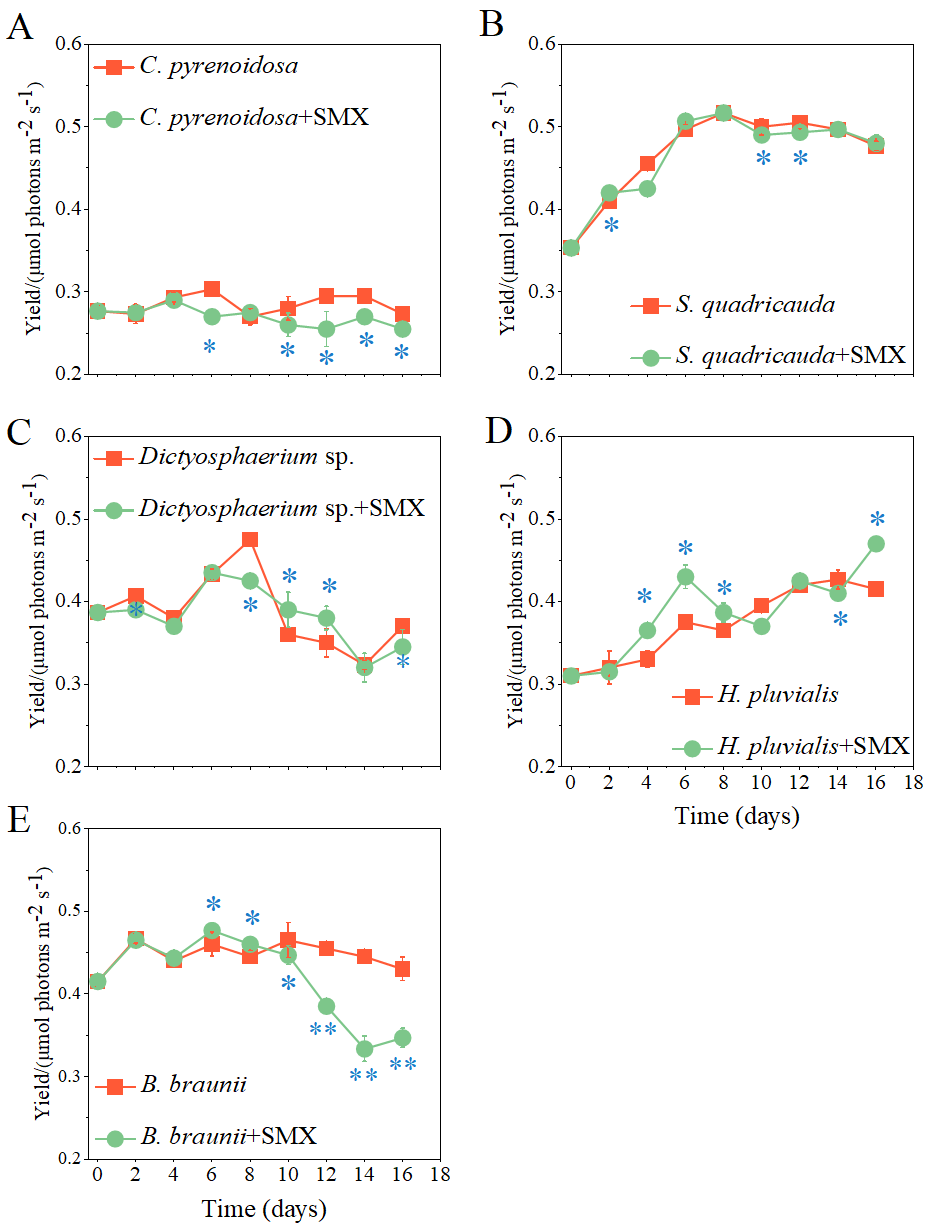


**Supplementary Figure 3.** Effects of SMX on the actual photosynthetic efficiency (*Yield*) of five microalgae (*C. pyrenoidosa*, *S. quadricauda*, *Dictyosphaerium* sp., *H. pluvialis* and *B. braunii*) during 16 days of cultivation. Error bars represent standard deviation (n=3). Asterisks indicate significant differences between the control and treatment group s (*p* < 0.05*; *p* < 0.01**).


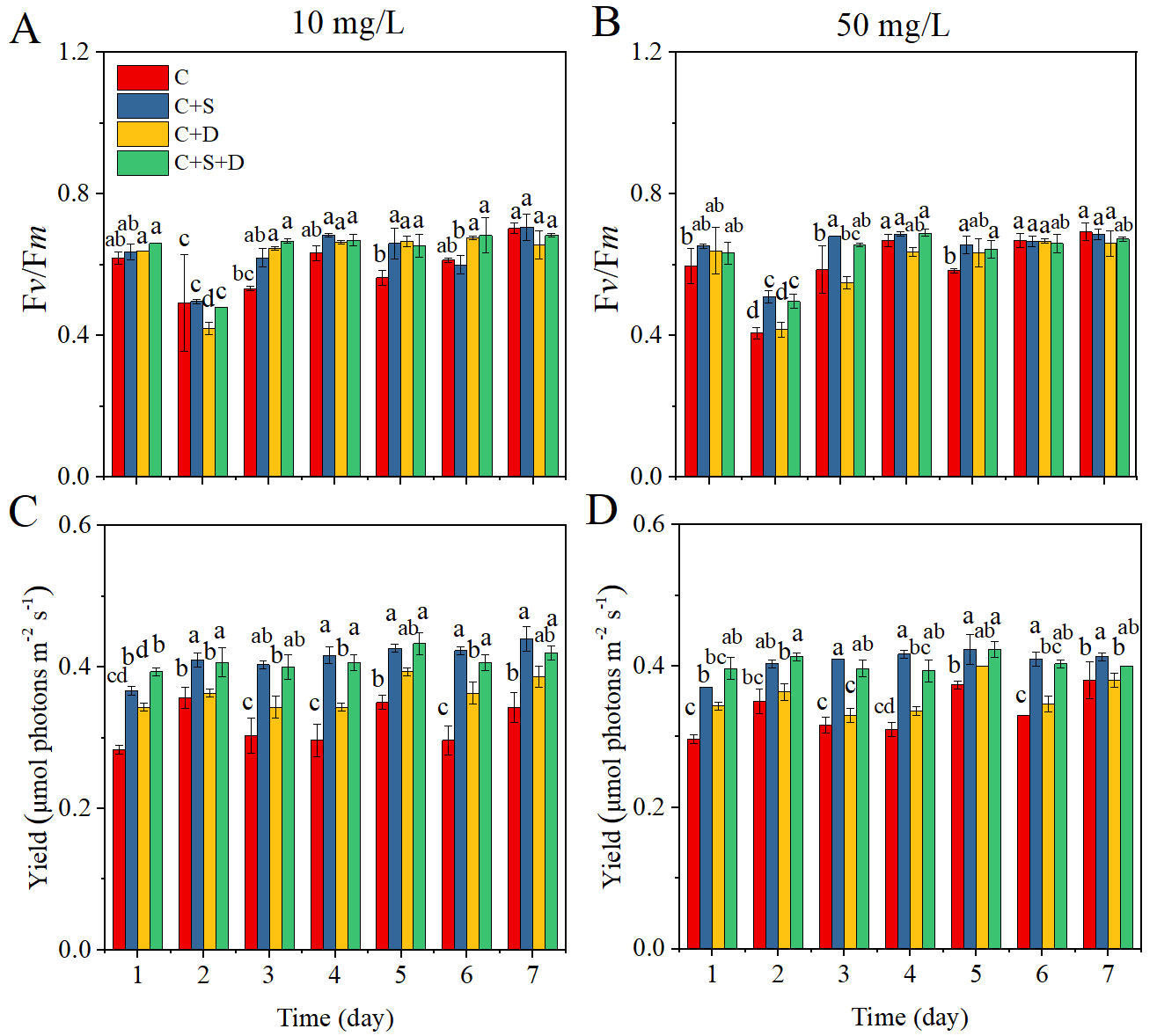


**Supplementary Figure 4.** Effects of the SMX on the maximum photosynthetic efficiency (*Fv/Fm*) and actual photosynthetic efficiency (*Yield*) of microalgae consortia during 7 days of cultivation. Error bars represent standard deviation (n=3). Columns with different letters indicate significant differences (*p* <0.05) between the control and treatment.
